# Research in etiology of Floppy Kid Syndrome

**DOI:** 10.3389/fvets.2025.1557951

**Published:** 2025-03-05

**Authors:** Cheng Cheng, Yan Zheng, Xin Wang, Jianping Tao, Darong Cheng

**Affiliations:** ^1^College of Veterinary Medicine, Yangzhou University, Yangzhou, Jiangsu, China; ^2^Jiangsu Co-innovation Center for Prevention and Control of Important Animal Infectious Diseases and Zoonoses, Yangzhou, Jiangsu, China

**Keywords:** goat, Floppy Kid Syndrome, etiology, metagenomics, microbiota

## Abstract

Floppy Kid Syndrome (FKS) is a common and serious disease in goats, with incidence rates ranging from 10 to 50% and mortality rates between 20 and 60%. This study aimed to investigate the etiology of FKS through blood biochemical analysis and metagenomic sequencing. Blood biochemical analysis revealed metabolic disorders in FKS-affected goats, including acidosis and hypoglycemia. Metagenomic analysis showed marked gastric and gut dysbacteriosis, characterized by an increase in pathogenic bacteria such as *Escherichia coli* and *Staphylococcus aureus*, alongside a significant reduction in probiotic like *Lactobacillus amylovorus*. Furthermore, species diversity and richness were notably lower in FKS-affected goats compared to healthy goats. Based on these findings, we infer that FKS is a multifactorial disease caused by gastric and gut dysbacteriosis. The immaturity of the digestive system in newborn goats, combined with environmental stressors (such as sudden changes in weather), leads to gastric and gut dysbacteriosis, with a significant reduction in probiotic and an overgrowth of pathogenic bacteria. The dysbacteriosis, along with the inability to properly digest excessive milk intake, contributes to the accumulation of undigested milk in the digestive tract, creating an environment conducive to pathogenic bacteria growth. The fermentation of milk and the production of excessive lactic acid by pathogenic bacteria are absorbed into the bloodstream, causing acidosis and hypoglycemia. These metabolic disorders, in conjunction with the dysbacteriosis and systemic dysfunction, lead to the onset of FKS. These results underscore the critical role of gastric and gut dysbacteriosis in the pathogenesis of FKS, highlighting the need for targeted preventive and therapeutic strategies.

## 1 Introduction

Floppy Kid Syndrome (FKS) is a serious disease affecting goats, this disease was described for the first time in the United States in 1987 and later in Canada and several countries in Europe (1). FKS primarily affects goat kids aged 4–15 days and occurs year-round, with a higher prevalence in goats born from January to April (2), with an incidence rate ranging from 10 to 50% and mortality rates reaching as high as 20 to 60% (3). Due to its complex etiology and the lack of effective prevention and treatment strategies, FKS poses a significant threat to the goat farming industry.

There is no consensus among researchers worldwide on the etiology of FKS. Some studies have shown that D-lactate levels in the blood of affected kids are significantly higher than in healthy ones, suggesting that the accumulation of D-lactate in the blood may induce metabolic acidosis, which could lead to FKS (4). However, sodium bicarbonate supplementation aimed solely at treating metabolic acidosis is generally ineffective in alleviating symptoms, indicating that acidosis may only be a part of the disease’s pathogenesis (5). Other studies propose that excessive milk intake and the overgrowth of pathogenic bacteria in the gut may be key contributing factors to the disease. This hypothesis explains why bicarbonate treatment alone does not yield satisfactory results, highlighting the need for targeted treatment of pathogenic bacteria in the gut. Some researchers have also suggested that mycotoxins might be one of the triggers for the disease (6), but this theory does not effectively explain the concentrated occurrence of the disease in winter and spring seasons.

In the early stages of FKS, goats typically exhibit symptoms such as depression and rapid heart rate. As the condition worsens, symptoms progress to delayed responses, slowed heart rate, and pale mucous membranes (7). FKS-affected goats may develop weakness in their limbs, unstable gait, abdominal distension, and vomiting. In severe cases, systemic flaccidity and limb spasms are observed (1). Anatomy of FKS-affected goats shows pulmonary edema with hemorrhagic spots, consolidation of the apical and cardiac lobes of the lungs, myocardial relaxation, and dilation of both ventricles. The abomasum is distended with a large amount of milk curd remnants, and the gastric contents have an acidic, foul odor. The abomasum may also be dilated and show hemorrhagic spots (8). The intestinal mucosa is congested and hemorrhagic, with yellow or gray-white milky, mucous substances remaining in the colon and rectum (9).

Currently, treatment for FKS mainly relies on symptomatic therapy, such as sodium bicarbonate for acidosis correction and glucose supplementation for hypoglycemia, but no specific medication is available. Some researchers suggest that separating ewes and lambs to prevent excessive milk intake may help reduce the incidence of the disease (10).

While existing research provides several hypotheses regarding the causes of FKS, studies on its microbiological basis are still in the early stages. Traditional research methods often rely on bacterial isolation and cultivation. However, due to the complex gastric and gut microbiota in the digestive tract and limited cultivation conditions, and many microorganisms are difficult to cultivate *in vitro*, it cannot fully reflect the true composition of gastric and gut microbiota (11). In recent years, metagenomics, as an emerging research method, has gained prominence. By enabling high-throughput analysis of the gut microbiota, metagenomics has become a powerful tool for investigating the relationship between diseases and microbial communities. This technique does not depend on traditional culture methods, allowing for a more comprehensive and efficient analysis of complex microbial communities, offering new perspectives and more precise data for studying the causes of complex diseases (12).

This study employs metagenomics to investigate the etiology of FKS, analyzing the differences in gastric and gut microbiota between FKS-affected goats and healthy goats. The findings aim to provide new insights into the microbiological mechanisms of the disease and offer a theoretical basis for future treatment and prevention strategies.

## 2 Materials and methods

### 2.1 Blood biochemical analysis

#### 2.1.1 Sample collection

Three healthy goats and five FKS-affected goats were randomly selected from the farm. The FKS-affected goats presented typical symptoms such as limb weakness and unstable gait. Blood samples (2–3 mL) were collected from the jugular vein of each goat via nonanticoagulant vacuum tubes. After collection, the blood samples were left at room temperature for 30 min to coagulate and then centrifuged at 3000 rpm to separate the serum. The serum was transferred to 1.5 mL centrifuge tubes, divided into TR1-TR5 (FKS-affected goats) and H1-H3 (healthy goats).

#### 2.1.2 Biochemical index measurement

Nine biochemical indices, including total protein, albumin, globulin, lactate dehydrogenase (LDH), creatine kinase (CK), glucose, calcium, phosphorus, and amylase, were measured via an automatic biochemical analyzer (Beckman Coulter International Trading Co., Ltd., Shanghai).

### 2.2 Gastric and gut microbiota analysis

#### 2.2.1 Sample collection

In this study, we analyzed the changes in the microbiota of the gastric and gut by collecting samples of the abomasum and duodenum from healthy goats and FKS-affected goats. Two healthy goats and seven FKS-affected goats were randomly selected from the farm. Goats in the FKS group were diagnosed based on clinical symptoms (limb weakness, unsteady gait) and confirmed by post-mortem examination, which revealed abomasum and duodenum flatulence. Healthy goats were confirmed post-mortem to have normal gastrointestinal morphology. The sampling procedures were as follows: Abomasum Content Collection: Samples were taken from the central lumen of the abomasum. Duodenum Content Collection: The duodenum was ligated at both ends (pylorus and jejunum) before excising a 10 cm segment, and luminal contents were gently extruded into sterile containers. All instruments were sterilized at 121°C before use. Samples were aliquoted into 1.5 mL sterile centrifuge tubes (≥1 g per tube), flash-frozen in liquid nitrogen within 5 min of collection, and stored at −80°C. The samples were divided into TR1S-TR7S (abomasum of FKS-affected goats), TR1I-TR7I (duodenum of FKS-affected goats), H1S-H2S (abomasum of healthy goats), and H1I-H2I (duodenum of healthy goats).

#### 2.2.2 Metagenomic sequencing

Each sample’s DNA (200 ng) was fragmented into 300--350 bp fragments via a Covaris S220 ultrasonicator. The DNA ends were repaired via the End Prep Enzyme Mix, and index adapters were added. After amplification via PCR with primers P5 (5’-AGATCGGAAGAGCGTCGTGTAGGGAAAGAGTGT-3′) and P7 (5’-GATCGGAAGAGCACACGTCTGAACTCCAGTCAC-3′), the products were sequenced via the Illumina HiSeq platform. Bcl2fastq software (v2.17.1.14) was used to process raw image data from the Illumina HiSeq platform into readable sequencing data (pass filter data). Sequencing adapters and low-quality bases (below 20) were removed via Cutadapt (v1.9.1), and sequences containing more than 10% N bases were discarded. Host DNA contamination was removed by aligning the reads to the host genome via BWA (v0.7.12), yielding clean data. On the basis of the clean data, a de Bruijn graph was constructed and assembled via MEGAHIT (v1.1.3). Gene prediction was performed via Prodigal (v3.02), and redundant sequences were removed via MMseqs2 (v2.0) with 95% sequence identity and coverage thresholds. Clean reads were mapped to the nonredundant gene set to calculate the relative abundance of each unigene in the samples. The unigene sequences were aligned against the NCBI NR database via Diamond software to obtain species annotation information. On the basis of the annotation results and gene abundance, the species abundance was calculated at different taxonomic levels (phylum, genus, and species).

## 3 Results

### 3.1 Blood biochemical analysis of FKS-affected goats

The results of blood biochemical analysis of healthy goats and FKS-affected goats revealed that the levels of blood protein, lactate dehydrogenase (LDH), creatine kinase (CK), calcium, phosphorus, glucose, and amylase in healthy goats were all within the normal range. In FKS-affected goats, some presented decreased levels of albumin, globulin, and total protein. All FKS-affected goats presented elevated levels of LDH and CK; most presented increased phosphorus levels, whereas all presented reduced levels of glucose and amylase (See Supplementary Table 1).

### 3.2 Gastric and gut microbiota analysis of FKS-affected goats

#### 3.2.1 Alpha diversity analysis

**Figure 1:**
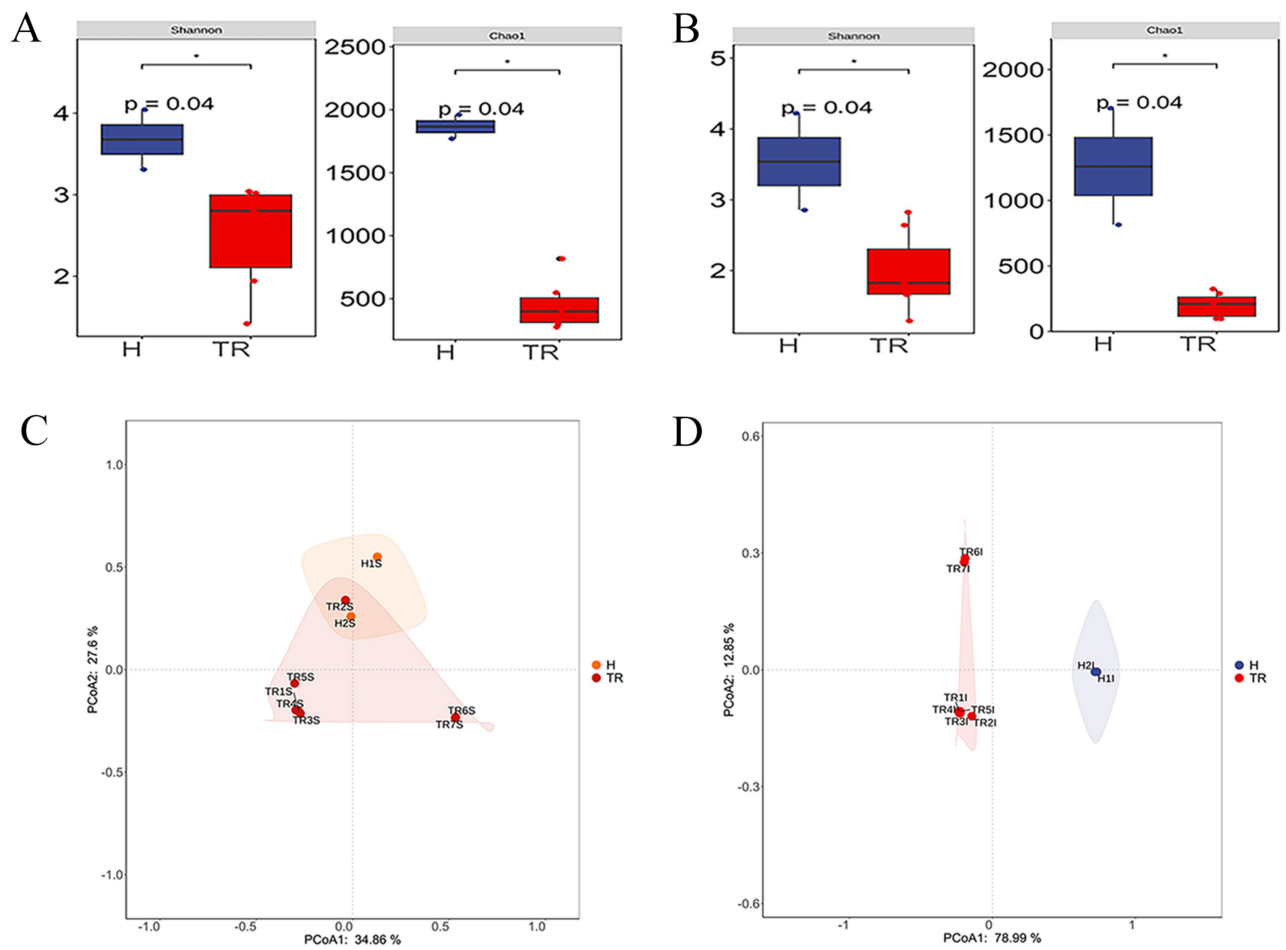
**(A)** Boxplot of alpha diversity index for abomasum; **(B)** Boxplot of alpha diversity index for duodenum; **(C)** Species composition PCoA diagram of abomasum; **(D)** Species composition PCoA diagram of duodenum.

Alpha diversity is used to analyze the diversity of microbial communities within a sample, including species richness, diversity, and evenness. The Shannon index estimates microbial diversity, with a higher Shannon index indicating greater diversity. The diversity of the abomasum and duodenum was significantly lower in the TR group than in the H group (*p* < 0.05) (Figures 1A,B). In both the abomasum and duodenum, the Chao1 index in the TR group was significantly lower than that in the H group (*p* < 0.05). These findings indicate that in both the abomasum and duodenum, the species diversity and richness of the TR group are lower than those of the H group (See Supplementary Table 2).

#### 3.2.2 Principal coordinate analysis

Principal coordinate analysis (PCoA) is a method used to visualize the similarities between samples, finding major differences through dimensionality reduction. PCoA1 and PCoA2 represent the percentage of variation explained by each dimension. In the abomasum group, the x-axis and y-axis explained 34.86 and 27.6% of the variation, respectively. Healthy goat samples (H1S ~ H2S) were clustered closely together. Except for TR2S, the remaining FKS samples were distant from the healthy group. Among the FKS group, TR1S, 3S, 4S, and 5S were closer to each other, whereas TR6 and 7S clustered together (Figure 1C). In the duodenum group, the x-axis and y-axis explained 78.99 and 12.85% of the variation, respectively. The healthy goat samples (H1I and H2I) were close to each other, whereas all the FKS samples were far from the healthy group. The FKS samples were concentrated on the left side of the x-axis, whereas the healthy group samples were concentrated on the right side. Within the FKS group, TR1I, 2I, 3I, 4I and 5I clustered closely, TR6I and 7I clustered closely. These findings indicate that the species compositions of the healthy and FKS groups are different (Figure 1D).

#### 3.2.3 Differences in species abundance and composition

**Figure 2:**
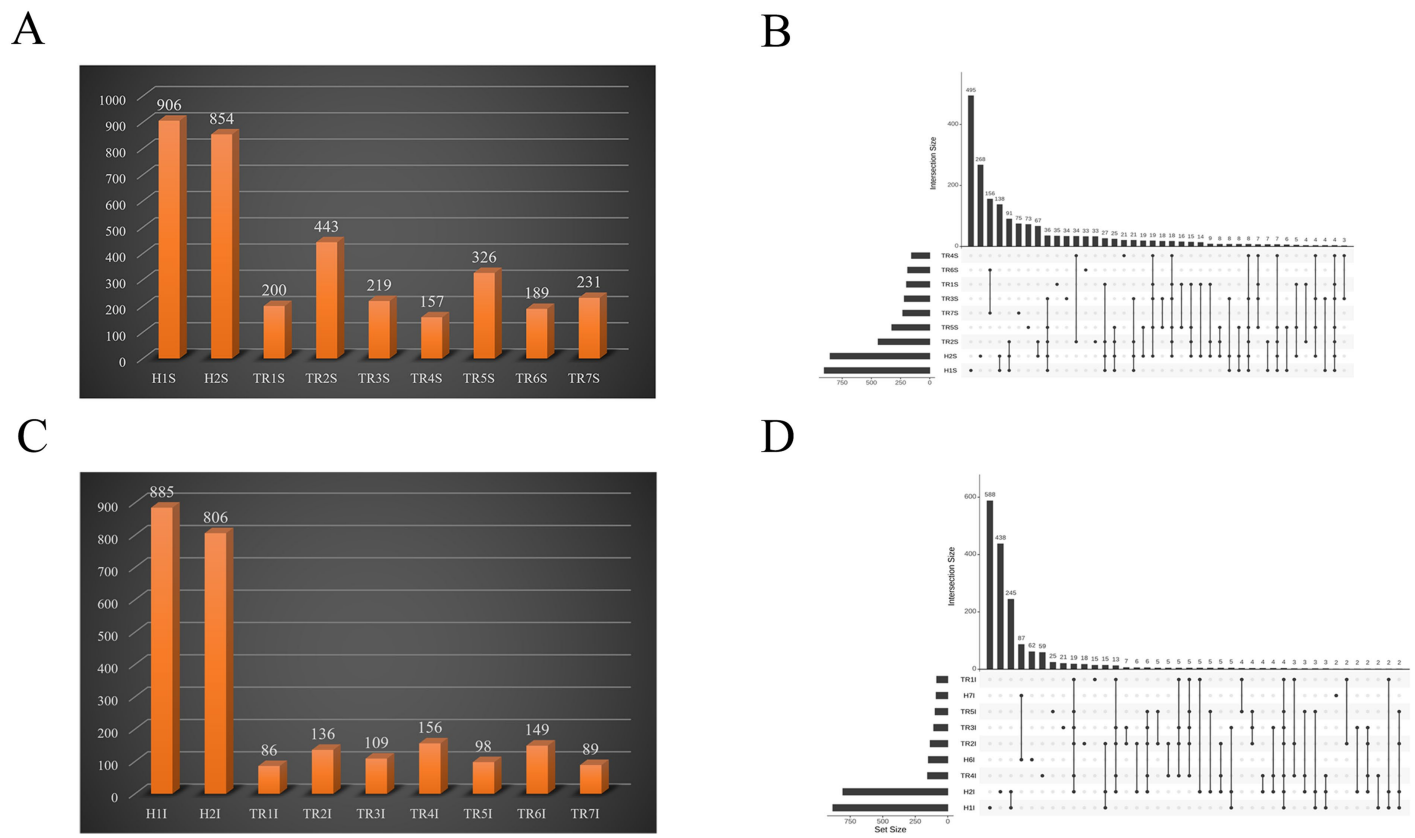
**(A)** Bar chart of species count of abomasum; **(B)** Species composition number upset diagram of abomasum; **(C)** Bar chart of species counts of duodenum; **(D)** Species composition number upset diagram of duodenum.

In the abomasum group, 906 and 854 species were healthy goats, whereas in the FKS goats, 200, 443, 219, 157, 326, 189, and 231 species were healthy goats, respectively. Compared with those of healthy goats, the differences in species abundance ranged from 411 to 749 species, with a significant reduction in the number of species in the abomasum of goats in the FKS (*p* < 0.05). The UpSet diagram shows that the number of unique species in FKS-affected goats also varied, with TR7S having the most unique species (75), followed by TR5S (73), TR1S (35), TR3S (34), TR2S and TR6S (33), and TR4S (13). The number of shared species between FKS-affected goats and healthy goats in the abomasum was less than 70. The most common species were between TR2S and H2S (67 species), whereas the least common species were between TR5S and H1S (6 species) (Figures 2A,B).

In the duodenum group, 885 and 805 species were healthy goats, whereas 86, 136, 109, 156, 98, 149, and 89 species were FKS goats. The difference in species abundance ranged from 650 to 799 species, with a significant reduction in the number of species in the duodenum of goats in the FKS treatment (*p* < 0.05). The number of unique species in the duodenum of FKS-affected goats was highest in TR6I (62 species), followed by TR4I (59 species), TR5I (25 species), TR3I (21 species), TR2I (18 species), TR1I (15 species), and TR7I (2 species). The number of shared species between FKS-affected goats and healthy goats in the duodenum was less than 10. The most common species were between TR2I and H2I (6 species), whereas the least common species were between TR4I and H1I (2 species). In summary, compared with healthy goats, FKS-affected goats presented a significantly lower number of species in both the abomasum and duodenum (*p* < 0.05), and the number of shared species was also significantly lower. Most of the species in the FKS-affected goats were newly added species. These findings indicate that the gastric and gut Microbiota of these goats had been disrupted, leading to reduced microbial diversity (Figures 2C,D).

#### 3.2.4 Relative abundance microbiota at the phylum level

**Figure 3:**
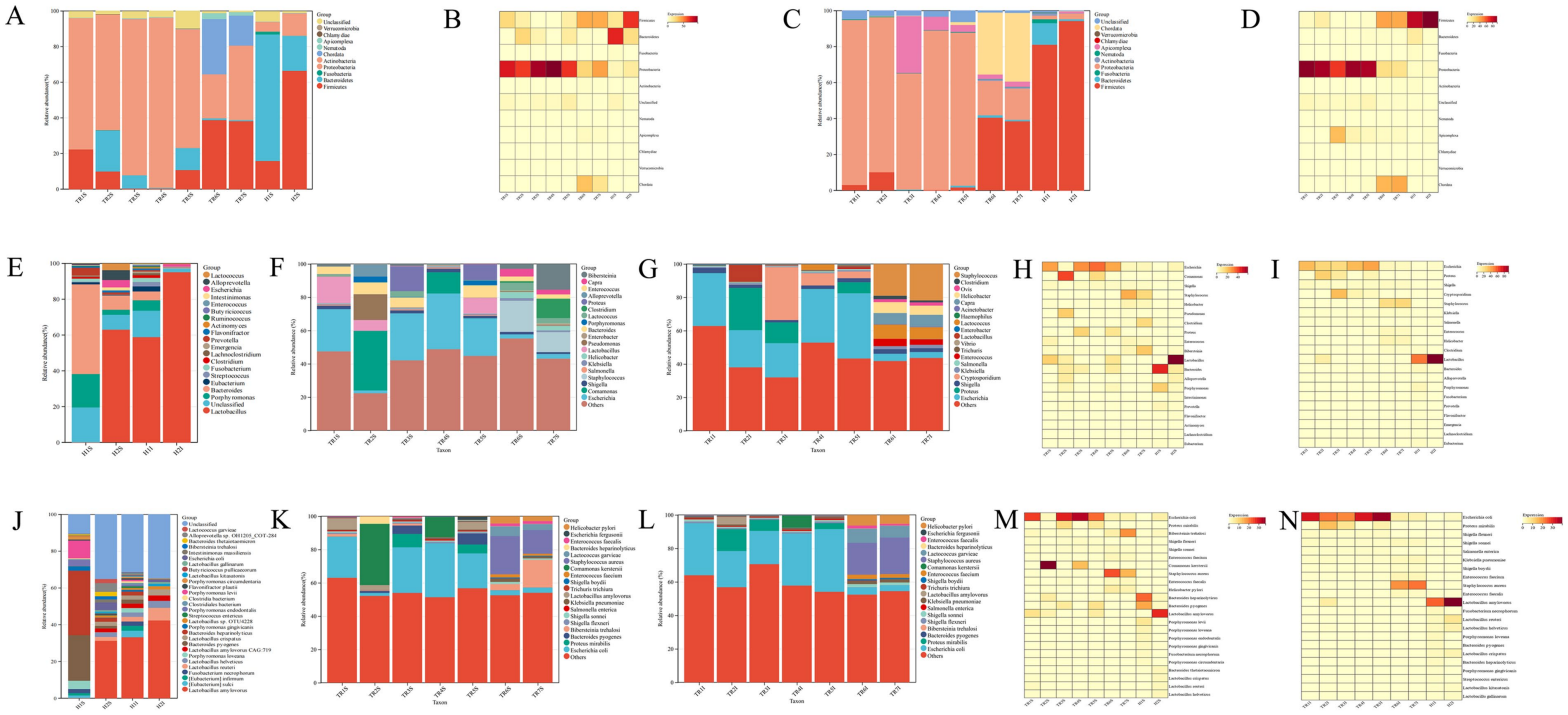
**(A)** The relative abundance of phylum level in abomasum; **(B)** Phylum-Level abundance heatmap in abomasum; **(C)** The relative abundance of phylum level in duodenum; **(D)** Phylum-Level abundance heatmap in duodenum; **(E)** The relative abundance of genus level of health goat; **(F)** The relative abundance of genus level in abomasum; **(G)** The relative abundance of genus level in duodenum; **(H)** Genus-Level abundance heatmap in abomasum; **(I)** Genus-Level abundance heatmap in duodenum; **(J)** The relative abundance of species level of health goat; **(K)** The relative abundance of species level in abomasum; **(L)** The relative abundance of species level in duodenum; **(M)** Species-Level abundance heatmap in abomasum; **(N)** Species-Level abundance heatmap in duodenum.

At the phylum level, the dominant phyla in the gastric and gut contents of healthy goats were Firmicutes and Bacteroidetes, with the combined relative abundance of these two phyla exceeding 85% in both the abomasum and duodenum samples. The relative abundance of Firmicutes was greater in the duodenum than in the abomasum, whereas Bacteroidetes had a greater relative abundance in the abomasum than in the duodenum. Proteobacteria was another phylum with a relative abundance of more than 1%, reaching 2.1% or more (See Supplementary Table 3). In abomasum of FKS-affected goats, compared with healthy goats, the relative abundances of Firmicutes (*p* < 0.05) and Bacteroidetes (*p* < 0.05) significantly decreased in the FKS-affected goats, whereas the relative abundance of Proteobacteria significantly increased (*p* < 0.05). Additionally, in TR6 and TR7S, the relative abundance of Chordata (*p* < 0.05) increased significantly (Figures 3A,B). In duodenum of FKS-affected goats, compared with healthy goats, the relative abundance of Firmicutes (*p* < 0.05) significantly decreased, whereas that of Proteobacteria significantly increased (*p* < 0.05). Furthermore, the relative abundance of Apicomplexa increased significantly in TR3I, and that of Chordata increased in TR6 and TR7I (Figures 3C,D).

#### 3.2.5 Relative abundance of microbiota at the genus level

The microbiota composition at the genus level differed slightly between healthy goats and healthy goats. The dominant genus in H1S was *Bacteroides*, followed by *Porphyromonas* and *Prevotella*. In H1I, H2S, and H2I, *Lactobacillus* was the dominant genus. The second most dominant genera in H1I and H2S were *Porphyromonas* and *Bacteroides*. In H2S, *Alloprevotella* and *Lactococcus* were also present, with relative abundances of 5.28 and 3.42%, respectively. In H2I, the relative abundance of *Lactobacillus* was extremely high, at 93.37%, with only *Escherichia* having a relative abundance greater than 1% (Figure 3E).

In abomasum of FKS-affected goats, the dominant genus in TR1S, TR3S, TR4S, and TR5S was *Escherichia*, with a relative abundance of more than 23.9%. In TR6 and TR7S, the dominant genus was *Staphylococcus*, with a relative abundance of more than 13.66%. In TR2S, the dominant genus was *Comamonas*, with a relative abundance of 38.48% (Figure 3F). In duodenum of FKS-affected goats, the dominant genus in TR1S, TR4S, and TR5I was *Escherichia*, with a relative abundance of more than 20.54%. In TR2I, the dominant genera were *proteus* and *Escherichia*, with relative abundances exceeding 20%. In TR3I, the most dominant genus was *Cryptosporidium*, with a relative abundance of 31.76%, followed by *Escherichia*, with a relative abundance of 20.54%. In TR6 and TR7I, *Staphylococcus* was the dominant genus, with a relative abundance of more than 19.07%, followed by *Lactococcus*, with a relative abundance of more than 7.06%. In TR2 and TR3I, *Proteus* also accounted for a certain proportion, with relative abundances of 25.32 and 12.45%, respectively (Figure 3G).

In abomasum of FKS-affected goats, compared with healthy goats, the genus whose relative abundance significantly increased was *escherichia* (*p* < 0.05), and other genera whose relative abundance notably increased included *Staphylococcus*, *Comamonas*, *Proteus*, *Clostridium*, and *Bibersteinia*. The relative abundance of *Staphylococcus* significantly increased in TR6 and TR7S (*p* < 0.05) but slightly increased in the other samples. The relative abundance of *Comamonas* increased substantially only in TR2S and TR4S, and that of *Proteus* increased in TR2S and TR5S. *Clostridium* and *Bibersteinia* only showed notable increases in TR7S (Figure 3H). In abomasum of FKS-affected goats, the genera whose relative abundance significantly decreased were *lactobacillus* and *Bacteroides*, whereas *Porphyromonas* slightly decreased. The decreases in other genera were not significant (See Supplementary Table 4). In duodenum of FKS-affected goats, compared with healthy goats, the genus that showed a significant increase in relative abundance was *Escherichia* (*p* < 0.05), whereas *Staphylococcus*, *Proteus*, *Cryptosporidium*, *Shigella*, *Enterococcus*, and *Helicobacter* showed more obvious increases. The relative abundance of *Staphylococcus* significantly increased in TR6 and TR7I (*p* < 0.05) but slightly increased in the other samples. *Proteus* showed a substantial increase in TR2I and slight increases in TR3 and TR5I. The relative abundance of *Cryptosporidium* substantially increased only in TR3I. The abundance of *Shigella* slightly increased in all the samples, and the abundances of *Enterococcus* and *Helicobacter* slightly increased only in TR6 and TR7I (Figure 3I). In duodenum of FKS-affected goats, the genus whose relative abundance significantly decreased was *Lactobacillus* (*p* < 0.05), whereas the relative abundances of *Bacteroides* and *Porphyromonas* slightly decreased (See Supplementary Table 4).

#### 3.2.6 Relative abomasum microbiota at the species level

At the species level, in healthy goats, the most dominant species in H1S were *Bacteroides pyogenes* and *Bacteroides heparinolyticus*, both with relative abundances above 15%. Other species with relative abundances exceeding 1% included *Porphyromonas loveana*, *Porphyromonas endodontalis*, *Porphyromonas levii*, *Porphyromonas circumdentaria*, and *Fusobacterium necrophorum*. In H1I, H2S, and H2I, the most dominant species was *Lactobacillus amylovorus*, with relative abundances exceeding 23%. Other species with relative abundances greater than 1% included *Lactobacillus reuteri*, *Lactobacillus helveticus*, *Lactobacillus crispatus*, *Bacteroides pyogenes*, and *Bacteroides heparinolyticus*. Additionally, in H1I, species such as *[Eubacterium] sulci*, *[Eubacterium] infirmum*, *Porphyromonas loveana*, and *Fusobacterium necrophorum* had relative abundances greater than 1%. In H2S, *Escherichia coli*, *Intestinimonas massiliensis*, *Bibersteinia trehalosi*, *Bacteroides thetaiotaomicron*, *Alloprevotella* spp., and *Lactococcus garvieae* presented relative abundances above 1%. In H2I, species such as *Lactobacillus kitasatonis*, *Lactobacillus gallinarum*, and *Escherichia coli* had relative abundances greater than 1% (Figure 3J).

In abomasum of FKS-affected goats, the dominant species in TR1S, TR3S, TR4S, and TR5S was *Escherichia coli*, with a relative abundance above 20.93%. In TR2S, the dominant species was *Comamonas kerstersii*, with a relative abundance of 36.75%. The dominant species in TR6S was *Staphylococcus aureus*, whereas TR7S was dominated by *Bibersteinia trehalosi* and *Staphylococcus aureus*, both with relative abundances exceeding 13.23% (Figure 3K). In duodenum of FKS-affected goats, the dominant species in TR1I, TR2I, TR3I, TR4I, and TR5I was *Escherichia coli*, with relative abundances exceeding 19.86%. In TR6 and TR7I, the dominant species was *Staphylococcus aureus*, whereas in TR2I, *proteus mirabilis* constituted a considerable proportion, with a relative abundance of 13.1% (Figure 3L).

In abomasum of FKS-affected goats, compared with healthy goats, the species whose relative abundance significantly decreased included *Bacteroides heparinolyticus*, *Bacteroides pyogenes*, and *Lactobacillus amylovorus*, all of which decreased by more than 15%. Other species, such as *Porphyromonas loveana*, *Porphyromonas gingivalis*, *Porphyromonas endodontalis*, *Porphyromonas levii*, *Lactobacillus reuteri*, *Lactobacillus helveticus*, and *Lactobacillus crispatus*, slightly decreased, ranging from 1 to 6% (Figure 3M). The species that presented a significant increase in relative abundance was *Escherichia coli* (*p* < 0.05), whereas other species with large increases included *Staphylococcus aureus*, *proteus mirabilis*, *Bibersteinia trehalosi*, and *Comamonas kerstersii*. The relative abundance of *Staphylococcus aureus* significantly increased in TR6 and TR7S (*p* < 0.05), with smaller increases in the other samples. The relative abundance of *Bibersteinia trehalosi* significantly increased only in TR7S, with a slight increase in TR6S. *Comamonas kerstersii* increased significantly in TR2 and TR3S. In addition, the relative abundances of *Proteus mirabilis* and *Helicobacter pylori* slightly increased in TR3 and TR5S and TR6 and TR7S, respectively (See Supplementary Table 5).

In duodenum of FKS-affected goats, compared with healthy goats, the relative abundance of *Lactobacillus amylovorus* significantly decreased (*p* < 0.05), with a decrease of more than 20%. Other species, such as *[Eubacterium] sulci*, *[Eubacterium] infirmum*, *Fusobacterium necrophorum*, *Lactobacillus reuteri*, *Lactobacillus helveticus*, *Lactobacillus crispatus*, *Lactobacillus kitasatonis*, *Bacteroides heparinolyticus*, *Bacteroides pyogenes*, and *Porphyromonas loveana*, showed relatively small decreases, ranging from 1 to 6.5% (Figure 3N). The species that presented a significant increase in relative abundance was *Escherichia coli* (*p* < 0.05). The relative abundance of *Staphylococcus aureus* significantly increased in TR6 and TR7I (*p* < 0.05), with slight increases in the other samples. The relative abundance of *proteus mirabilis* slightly increased in TR1, TR2, and TR4I, whereas *Comamonas kerstersii* significantly increased, exceeding 7% in TR4I. The relative abundances of *Helicobacter pylori*, *Enterococcus faecium*, *Shigella sonnei*, and *Klebsiella pneumoniae* slightly increased in TR6 and TR7I, but the increases in *Helicobacter pylori* did not exceed 6.5%, and the increases in *Enterococcus faecium*, *Shigella sonnei*, and *Klebsiella pneumoniae* did not exceed 3% (See Supplementary Table 5).

### 3.3 Correlation analysis between blood biochemical indicators and microbiota

Elevated lactate dehydrogenase (LDH) and creatine kinase (CK) levels in FKS-affected goats were significantly correlated with the overgrowth of *Escherichia coli* (*p* < 0.01) and *Staphylococcus aureus* (*p* < 0.05). Severe hypoglycemia was significantly and inversely correlated with the abundance of *Lactobacillus amylovorus* (*p* < 0.001) and significantly correlated with the increased abundances of *Escherichia coli* (*p* < 0.01) and *Staphylococcus aureus* (*p* < 0.05). Additionally, hyperphosphatemia was correlated with the abundance of *Proteus mirabilis* (*p* < 0.05), while hypoproteinemia was correlated with the reduction of *Bacteroides heparinolyticus* (*p* < 0.01) and the overgrowth of *Clostridium* spp. (*p* < 0.05). A reduction in overall *α*-diversity was also observed in FKS-affected goats. These results demonstrate a direct link between dysbacteriosis and metabolic disorders in FKS pathogenesis (Table 1).

**Table 1:** Correlation analysis between blood biochemical indicators and microbiota.

Biochemical indicator	Microbiota taxon	Correlation	*p*-value
Lactate Dehydrogenase (LDH)	*Escherichia coli*	Positive	*p* < 0.01
Creatine Kinase (CK)	*Staphylococcus aureus*	Positive	*p* < 0.05
Glucose (GLU)	*Lactobacillus amylovorus*	Negative	*p* < 0.001
Glucose (GLU)	*Escherichia coli*	Positive	*p* < 0.01
Glucose (GLU)	*Staphylococcus aureus*	Positive	*p* < 0.05
Phosphorus (P)	*Proteus mirabilis*	Positive	*p* < 0.05
Total Protein (TP)	*Bacteroides heparinolyticus*	Negative	*p* < 0.01
Total Protein (TP)	*Clostridium* spp.	Positive	*p* < 0.05

## 4 Discussion

Floppy Kid Syndrome is a multifactorial disease characterized by metabolic disorders and systemic dysfunction in newborn goats. Our study provides novel insights into the role of gastric and gut dysbiosis in FKS pathogenesis, demonstrating a direct link between dysbiosis and blood biochemical abnormalities. The integration of metagenomic sequencing and blood biochemical analysis reveals a complex interplay between pathogenic bacteria overgrowth, loss of probiotics, and metabolic disorders.

Blood biochemical analysis revealed significantly elevated levels of lactate dehydrogenase (LDH) and creatine kinase (CK) in FKS-affected goats. The marked increase in LDH indicates that acidosis is closely linked to the overgrowth of *Escherichia coli*, which ferments undigested lactose to produce excessive lactic acid and releases toxins such as *Escherichia coli* lipopolysaccharide and *Staphylococcus aureus α*-hemolysin that led to symptoms worsen (14–17). Concurrent hyperphosphatemia was linked to *Proteus mirabilis* abundance, suggesting bacterial phosphatase activity and renal dysfunction exacerbate phosphate accumulation (18, 19). These findings align with previous reports that metabolic acidosis impairs myocardial function and electrolyte balance (20). The high abundance of *Escherichia coli* and *Staphylococcus aureus* aggressively consume glucose, thereby depleting its availability in the bloodstream. Therefore, severe hypoglycemia was observed and is predominantly attributed to this intense pathogenic bacteria glucose consumption, compounded by the significant reduction in *Lactobacillus amylovorus*—a key probiotic that normally promotes glucose uptake via the upregulation of intestinal SGLT1 (13). The marked decline in *Lactobacillus amylovorus* further exacerbates the low blood glucose state, as its diminished presence undermines the host’s capacity for efficient nutrient absorption. Additionally, the marked reduction in pancreatic amylase further reflects *Staphylococcus aureus*-mediated inhibition of exocrine pancreatic function (21). These results robustly support the hypothesis that dysbiosis disrupts glucose homeostasis through a dual mechanism: dominant pathogenic bacteria intensively competing for and metabolizing glucose, and the loss of beneficial probiotic populations impairing nutrient absorption, ultimately leading to the hypoglycemic and acidosis characteristic of FKS.

Comparative metagenomic analysis revealed marked alterations in the microbial composition of the gastric and gut in FKS-affected goats, characterized by a significant increase in pathogenic bacteria such as *Escherichia coli*, *Staphylococcus aureus*, *proteus*, and *shigella*, with *Escherichia coli* and *Staphylococcus aureus* showing the most significant increases. Numerous studies suggest that an increase in these bacteria contributes to diarrhea and gastric and gut dysbacteriosis (22, 23). At the same time, probiotic such as *Lactobacillus amylovorus*, *Bacteroides heparinolyticus*, *Bacteroides pyogenes*, and others decreased, with *Lactobacillus amylovorus* showing the most significant decreases. Research indicates that *Lactobacillus* and *Bacteroides* contribute to maintaining a healthy gastric and gut microbiome, inhibiting the growth of pathogens, boosting immunity, and improving feed conversion efficiency (24–26). A marked decrease in these probiotics may result in lowered immunity, impaired gastric and gut barrier function, and related complications. Certain studies propose a connection between probiotic levels and digestive efficiency. Probiotics aid in breaking down fats and organic matter, facilitating digestion and enhancing nutrient absorption (27). The marked reduction in probiotics in FKS-affected goats likely diminishes digestive capacity, exacerbating the condition.

Synthesizing the result of blood biochemistry and metagenomic, we infer that FKS has a multifaceted etiology: newborn goats, with underdeveloped digestive systems, experience reduced gastric acid and pepsin secretion and slower intestinal motility, hindering the elimination of pathogens. Sudden weather changes that stress or chill goats may exacerbate gastric and gut dysbacteriosis, leading to a substantial loss of probiotics and a compromised intestinal barrier, making the gastric and gut more susceptible to pathogenic invasion and proliferation. When goats with inadequate digestive capacity consume excess milk, undigested milk accumulates in the digestive tract, fostering an ideal environment for pathogenic bacterial growth. Consequently, pathogenic bacteria multiply in the gastric and gut, and fermentation of the accumulated milk, combined with bacterial breakdown of lactose, generates large amounts of lactic acid. Due to the goat’s weak digestive system, this lactic acid is excessively absorbed into the bloodstream, while the increasing abundance of *Escherichia coli* and *Staphylococcus aureus* consume glucose, and the decrease in *Lactobacillus amylovorus* abundance hinders glucose absorption, resulting in acidosis and hypoglycemia. The combination of increase in pathogenic bacteria abundance and decrease in probiotic abundance, leads to symptoms of acidosis and hypoglycemia, finally resulting in the development of FKS. Zheng et al. suggest that the occurrence of FKS is linked to sudden weather changes, poor farm management, and the congenital weakness of newborn goats, which lowers their resistance, enabling pathogenic bacteria such as *Escherichia coli* to overgrow and trigger disease (28). Chang et al. argue that FKS arises from disrupted abomasum function, leading to milk accumulation and excessive lactic acid production, causing acidosis and secondary bacterial infections, ultimately resulting in the goats’ weakness (29). Liu et al. propose that the onset of FKS is associated with the health of the ewe, with poor nutritional content in the milk contributing to the goats’ condition (30). The findings of these studies bear certain similarities to the results of this study, the analysis of the gastric and gut microbiota in FKS-affected goats revealed severe dysbacteriosis compared to healthy goats, specifically a marked decrease in species diversity, an abnormal increase in pathogenic bacteria like *Escherichia coli* and *Staphylococcus aureus*, and a reduction in probiotic such as *Lactobacillus amylovorus*. In summary, the gastric and gut dysbacteriosis, along with an abnormal increase in specific pathogenic bacteria, may be the main contributing factors to the onset of FKS in goats.

To further deepen the understanding of the etiology of FKS, future research should focus on several key areas. First, while this study revealed changes in the abundance of pathogenic bacteria associated with FKS, pathogen isolation and virulence factor analysis were not conducted. Future studies should prioritize isolating pathogens from FKS-affected goats, identifying their virulence genes, and evaluating their pathogenicity through antibiotic susceptibility testing, which will provide scientific evidence for clinical treatment. Second, although microbial changes in the gastric and gut microbiota of FKS-affected goats were analyzed, the specific mechanisms by which microbial interactions contribute to dysbacteriosis have not been fully explored. Future research should investigate the interactions between microorganisms and how they may exacerbate FKS by either inhibiting probiotic or promoting the growth of pathogenic bacteria. Additionally, the potential therapeutic role of probiotics like *Lactobacillus amylovorus* in FKS should be further explored. Clinical trials on probiotics are necessary to evaluate their effectiveness in restoring gut microbiota balance and improving FKS treatment. Furthermore, while this study suggests that weather changes may trigger FKS outbreaks, the specific environmental factors, such as temperature and humidity fluctuations, have not been thoroughly examined. Future studies should investigate the influence of these environmental variables on the gut microbiota and disease development. By addressing these areas, future research will provide a more comprehensive understanding of FKS’s complex etiology and offer more precise scientific evidence for its clinical treatment and prevention.

## 5 Conclusion

In summary, this study identifies key biochemical and microbial changes associated with FKS in goats, including metabolic acidosis, hypoglycemia, and dysbacteriosis of the gastric and gut microbiota. The findings infer that FKS is a multifactorial disease caused by gastric and gut dysbacteriosis. These results highlight the need for further research on pathogen isolation, virulence factor analysis, and the potential therapeutic role of probiotics. Understanding the complex etiology of FKS will aid in developing effective treatments and prevention strategies.

## Data Availability

The datasets presented in this study can be found in online repositories. The names of the repository/repositories and accession number(s) can be found below: https://www.ncbi.nlm.nih.gov/genbank/, BioProject ID PRJNA1228333.
